# New-Onset Systemic Capillary Leak Syndrome in an Adult Patient with COVID-19

**DOI:** 10.1155/2021/8098942

**Published:** 2021-09-30

**Authors:** Daniel B. Knox, Vivian Lee, Lindsay Leither, Samuel M. Brown

**Affiliations:** ^1^Pulmonary and Critical Care Medicine, Intermountain Medical Center, Murray, UT, USA; ^2^Pulmonary and Critical Care Medicine, University of Utah, Salt Lake City, UT, USA

## Abstract

Systemic capillary leak syndrome (SCLS) is a rare disorder manifesting as shock, hemoconcentration, and hypoalbuminemia, which may be either idiopathic or secondary to an underlying disease process. We report a case of an adult with COVID-19 infection associated with new onset SCLS. Our case joins only two other cases of new SCLS associated with COVID-19 infection reported in the literature. The clinical and laboratory findings in this case are distinct from findings seen in COVID-19 cytokine storm syndrome. Whether our therapeutic approach was efficacious is unknown. Further research into causes and therapies for these syndromes is urgently indicated.

## 1. Introduction

Systemic capillary leak syndrome (SCLS) is a rare and often fatal disease, manifested as transient but often recurrent episodes of shock, anasarca, hypoalbuminemia, and hemoconcentration [1]. Commonly identifiable triggers include viral infections, extended travel, and vigorous physical exertion [2]. In his original description of the disorder, Clarkson demonstrated that albumin-binding dye (Evans Blue) quickly extravasated from the intravascular space, suggesting vascular endothelial hyperpermeability as the primary disorder [3]. Complications of SCLS include renal failure, pericardial effusions/tamponade, and vascular thrombosis/pulmonary embolism resulting from hemoconcentration and subsequent hyperviscosity [2]. Crystalloid given during the acute leak phase often results in death secondary pulmonary edema in the postleak phase [4]. Severe peripheral anasarca may cause limb compartment syndromes necessitating fasciotomies and limb amputations. It can lead to severe, permanent disability secondary to senosorimotor neuropathy [2]. Death may occur during the postleak phase as a result of pulmonary edema [1, 4]. The precise pathophysiology of SCLS is still uncertain but is felt to involve inflammation resulting from circulating humoral factors such as IL-6, CXCL-10, VEGF, angiopoietin-2, CCL2, and transient vascular endothelial dysfunction [5–7]. In SCLS, 80-95% of adults have a monoclonal protein, typically small ~5 g/L, typically IgGk, whereas those <20 years typically do not have an M-protein (Pineton [8]).

COVID-19 is also associated with increased markers of inflammation, vascular endothelial dysfunction, and deleterious cytokine storm ([9–12]). We report a case of new onset SCLS in an adult patient with a history of mild COVID-19 symptoms and no other predisposing factor for SCLS. This case demonstrates salient clinical features seen in SCLS including refractory shock, multiple organ failure, hemoconcentration, pericardial effusion, and venous thromboses. To date, there are only two other reports of new onset SCLS associated with COVID-19 [13, 14]. Of the two reported, only one has survived to date [14]. The case described here adds to the existing body of knowledge surrounding COVID-19-associated SCLS and the range of therapies administered. There are three different etiologies of COVID-19-associated SCLS in the literature to date: (1) new onset [13, 14], (2) exacerbations of preexisting SCLS (de [15]), and (3) vaccine-induced SCLS [16, 17].

## 2. Case Presentation

A 48-year-old mildly asthmatic female presented to an outside hospital with complaints of vomiting, weakness, and syncopal symptoms. Nine days prior, she had self-limited symptoms of upper respiratory infection, anosmia, and dysgeusia, which she managed without medical attention. She tested positive for COVID-19 two days prior to admission. Upon admission, she was hypotensive, with mottled extremities. 6 L crystalloid was infused, and an echocardiogram was obtained, showing pericardial effusion, low-normal left ventricular function, no pulmonary hypertension, and normal right ventricular function. She was transferred to our quaternary care hospital for management of presumed impending cardiac tamponade. Upon arrival to our hospital, the volume of pericardial fluid had decreased substantially but was sampled under ultrasound guidance, demonstrating a nonspecific inflammatory profile (Table 1). The EKG was also nonspecific. She was treated with glucocorticoids. She was also treated for presumptive myocarditis and with broad spectrum antibiotics for possible sepsis. She had a clear chest X-ray and no respiratory symptoms. She manifested ongoing hypovolemic shock with purple extremities, worsening hemoconcentration, anuria, and central venous pressure < 5 cm H2O despite rapid infusion of 9 liters of crystalloid solution. Inflammatory markers were low: CRP and IL-6 were normal, while ferritin and fibrinogen were minimally elevated. Extensive serological workup including tryptase levels, C1 esterase levels, and serum electrophoresis revealed no alternative or contributing diagnosis, and all microbiological cultures were negative.

Her clinical course was consistent with systemic capillary leak syndrome (SCLS), and she was treated with high-dose IVIG, high-dose vasopressors, continuous renal replacement therapy, remdesivir, and a single dose of vitamin B12 for refractory vasoplegia. Heparin was started for widespread deep venous thromboses. Following IVIG administration, she was able to be weaned off multiple high-dose vasopressors within three hours. Her only hypoxemia occurred at resolution of vasoplegia and responded promptly to removal of excess fluid through continuous renal replacement therapy.

Upon discharge from the hospital a week following admission, the patient was ambulatory and normoxic, and her multiple organ function had completely recovered. Upon outpatient follow-up, the patient consulted with the National Institutes of Health (NIH) expert clinicians to be enrolled in a clinical study cohort and was considering further outpatient therapies.

## 3. Discussion

To our knowledge, there have been only two previously reported cases of *new* onset SCLS associated with COVID-19. One was a 63-year-old man with a history of hypertension who developed COVID-19-induced SCLS, leading to severe hypovolemic shock, multiple organ failure, 4-limb compartment syndrome, and death [13]. Another was a 38-year-old previously healthy male who developed COVID-19-associated SCLS, developed hypovolemic shock, multiple organ failure, and survived [14]. Autopsy data suggests an endotheliopathy in some patients who die from COVID-19 which may be related to the physiology observed in our patient [9, 10]. We anticipate that more similar cases in adults will be identified. It is unclear whether the pathophysiology mirrors the pediatric syndrome of idiopathic “toxic shock”-like syndrome reported by Feldstein and colleagues or whether it represents an entirely separate process [18]. Notably, unlike the syndrome described in children, our patient had only minimal markers of inflammation. There is current thoughts that in COVID-19 cytokine storm syndrome IL-6 plays a central role [19]. In SCLS, there appears to be low IL-6 and CRP initially which appears to separate these two diseases [15]. Whether our therapeutic approach—IVIG, steroids, remdesivir, CRRT, and heparin—was efficacious is unknown. Further research into causes and therapies for these syndromes is urgently indicated.

## Figures and Tables

**Table 1:** 

	Day 1 (night of admission)	Day 2	Day 3	Day 4	Day 5	Day 6	Day 7	Day 8
Hemoglobin (g/dL)	16.4–19.4	21.1-17.8	10.9-14.6	7.9-9.7	7.9-8.2	7.4-8.0	7.3	8.0
Albumin	1.0-3.0	2.2-2.6	2.5-2.6	2.7	2.9	—	3.4	3.6
Total crystalloid infused	9 liters	0	0	0	0	0	0	0
Total albumin infused	12.5 gm	50 gm	12.5 gm	0	0	0	0	0
IVIG		40 gm	40 gm	40 gm	40 gm	40 gm	40 gm	—
Shock present	+	+/- resolved by 14 : 30	—	—	—	—	—	—
Renal failure present	+	+	+	+	+	+	+	—
CRRT	—	+	+	+	+	+	+	—

## Data Availability

In order to protect patient privacy and comply with relevant regulations, identified data are unavailable. Requests for deidentified data from qualified researchers with appropriate ethics board approvals and relevant data use agreements will be processed by the Intermountain Office of Research, officeofresearch@imail.org.
